# Symptom Relief and Practice Setting Variation in Bulkamid Injections for Stress Urinary Incontinence

**DOI:** 10.1002/nau.70289

**Published:** 2026-04-09

**Authors:** Christina Sze, Dhillon Advano, Carolina Martinez Fernandez, Maali LaFrance, Maude Carmel, Ramy Goueli, Gary E. Lemack

**Affiliations:** ^1^ Department of Urology University of Texas Southwestern Medical Center Dallas Texas USA; ^2^ University of Texas Southwestern Medical School Dallas Texas USA

## Abstract

**Introduction:**

Bulkamid™ transurethral injection is a minimally invasive treatment for stress urinary incontinence (SUI) and mixed urinary incontinence (MUI). While effective, predictors of repeat injection, progression to sling, and the influence of anesthesia type on treatment efficacy remain incompletely defined.

**Methods:**

We retrospectively reviewed 284 women who underwent Bulkamid™ injection for SUI/MUI. Baseline demographics, comorbidities, and symptom scores were collected. Outcomes were assessed with paired Wilcoxon signed‐rank tests. Univariate logistic regression identified predictors of repeat injection and progression to sling. Subgroup analysis compared outcomes by anesthesia use.

**Results:**

The cohort had a median age of 63.5 years (IQR 50–72) and BMI of 28.3 kg/m² (24.0–31.7). Most were White (82.4%), with 77.8% having SUI and 22.2% MUI. Comorbidities included diabetes (21.1%), hypertension (48.2%), and prior hysterectomy (44.0%). Median follow‐up was 6.7 months (IQR 1.1–17.4). Bulkamid™ significantly improved continence and quality‐of‐life measures. Median pad use decreased from 2.0 to 1.0 per day (*p* < 0.001). UDI‐6 total scores declined from 45.8 to 16.7 (*p* < 0.001), with improvements in frequent urination, urgency leakage, physical activity leakage, difficulty emptying, and pain/discomfort (all *p* < 0.001). Comparison by anesthesia type (general/sedation vs none) showed similar pad reduction and UDI‐6 score improvements, but differences in voiding outcomes. Symptom score improvements were otherwise comparable, though those without anesthesia had shorter UDI‐6 follow‐up intervals (84 vs. 218 days, *p* = 0.002) and longer overall follow‐up (13.4 vs. 6.2 months, *p* = 0.016). On logistic regression, repeat injection was required in 16.5% at a median of 7.6 months, with procedure done without general/sedative anesthesia as strong predicator for repeat injection (OR 2.82, 95% CI 1.33–5.79, *p* = 0.005). Seventeen patients (6.0%) progressed to sling, with younger age predicting progression (OR 0.95 per year, 95% CI 0.91–0.98, *p* = 0.007).

**Conclusions:**

Bulkamid™ injection is a safe, effective, and minimally invasive treatment for stress and mixed urinary incontinence, offering significant symptom improvement with low rates of repeat injection and sling conversion. Younger age was associated with progression to sling, while procedures performed without general/sedative anesthesia were associated with higher likelihood of repeat injection. Bulkamid™ remains a valuable option for women seeking alternatives to sling surgery.

## 1 Introduction

Stress urinary incontinence (SUI) is a common condition affecting up to one in three women, with significant impact on quality of life, daily functioning, and psychosocial well‐being. Mixed urinary incontinence (MUI), in which patients experience both stress and urgency leakage, represents an additional therapeutic challenge. Midurethral sling (MUS) placement is the gold standard for refractory SUI, however, slings are associated with risks such as mesh related complications, post‐operative voiding dysfunction and need for revision surgery [1, 2].

Urethral bulking agents have emerged as a minimally invasive alternative for women seeking treatment outside of sling surgery. Bulkamid™, a polyacrylamide hydrogel, has gained wide adoption due to its biocompatibility, durability, and ease of administration in the office setting since its approval by the FDA in 2020 [3]. Clinical studies have demonstrated meaningful improvements in incontinence symptoms and quality of life following Bulkamid™ injection, with a favorable safety profile and low risk of urinary retention [4]. However, durability of effect, predictors of repeat injection, and factors associated with progression to sling remain less well defined [3, 5]. Furthermore, while Bulkamid™ can be performed with or without anesthesia, data comparing outcomes by anesthesia type are limited [6, 7].

The objective of this study was to evaluate outcomes of Bulkamid™ injection at a single institution across multiple surgeons. Specifically, we aimed to assess improvements in patient‐reported and objective measures of continence using daytime pad usage, identify predictors of repeat injection and progression to sling, and compare outcomes between patients treated with and without anesthesia.

## 2 Methods

We conducted a retrospective review of women who underwent urethral bulking with Bulkamid™ at a single academic institution between 2018 and 2024. Procedures were performed by multiple attending urologists and female pelvic medicine specialists. Bulkamid™ injections were performed either in the operating room with intravenous sedation (with anesthesia) or in the outpatient clinic setting without intravenous anesthesia (without anesthesia). All clinic‐based procedures were performed using intraurethral lidocaine jelly for topical anesthesia. Patients with a history of prior midurethral sling placement or other prior urethral bulking procedures were excluded from analysis.

Demographic and clinical data were abstracted from the electronic medical record, including age, race, body mass index (BMI), comorbidities (diabetes mellitus, hypertension), history of hysterectomy, smoking status, and concurrent use of anticholinergic or beta‐agonist medications. Urodynamic and post‐void residual (PVR) data were recorded when available.

Primary outcomes included changes in pad usage during waking hours, PVR, and validated patient‐reported outcome measures (Urogenital Distress Inventory [UDI‐6] subdomains and total score) before and after Bulkamid™ injection. Secondary outcomes included the need for repeat injection, progression to sling surgery, and comparisons of outcomes by anesthesia type.

In our practice, UDI‐6 forms are routinely administered prior to each clinic visit, however, completion is inconsistent, and not all patients have this data available. Post‐operative follow‐up is typically scheduled at approximately 6 weeks after the procedure, though actual visit timing varies in routine clinical practice. Decisions regarding repeat Bulkamid™ injections were driven by patient‐reported symptom control and satisfaction. Repeat injections were initiated either at post‐operative follow‐up visits or when patients independently contacted the clinic due to persistent or worsening symptoms.

Pre‐ and post‐intervention outcomes were compared using paired Wilcoxon signed‐rank tests. Between‐group comparisons (e.g., anesthesia type) were assessed using Wilcoxon rank‐sum tests. Univariate logistic regression was performed to identify predictors of repeat injection and progression to sling surgery. Odds ratios (OR) with 95% confidence intervals (CI) were reported. A two‐tailed *p*‐value < 0.05 was considered statistically significant. Analyses were performed using R version 4.4.3.

## 3 Results

A total of 284 women underwent Bulkamid™ injection with a median follow‐up of 6.7 months (IQR 1.1–17.4). The median age was 63.5 years (interquartile range [IQR] 50.0–72.0) and the median body mass index (BMI) was 28.3 kg/m² (IQR 24.0–31.7). The majority were White (82.4%), with smaller proportions identifying as Black (6.0%), Other (5.6%), or Unknown (6.0%). Based on history most patients carried a diagnosis of stress urinary incontinence (SUI) (77.8%), while 22.2% had mixed urinary incontinence (MUI). Comorbidities included diabetes mellitus (DM) in 21.1%, hypertension (HTN) in 48.2%, and prior hysterectomy in 44.0% of patients. Regarding smoking status, 71.1% were never smokers, 25.4% were former smokers, and 3.6% were current smokers. Anticholinergic use was present in 24.7% and beta‐agonist use in 33.1% of patients (Table 1). Median total UDI‐6 score preprocedural was 45.8 (IQR 33.3–58.3), and median total number of Bulkamid™ injections was 1 (1‐1).

**Table 1:** Patient demographics & clinical characteristics.

Characteristic	*N* = 284
Age, years, median (IQR)	63.50 (50.00–72.00)
Race	
White	234/284 (82.39%)
Black	17/284 (5.99%)
Other	16/284 (5.63%)
BMI (kg/m^2^)	28.29 (24.03–31.72)
SUI Diagnosis	221/284 (77.82%)
MUI Diagnosis	63/284 (22.18%)
Smoking Status	
Never	199/280 (71.07%)
Former	71/280 (25.36%)
Current	10/280 (3.57%)
Prior Hysterectomy	125/284 (44.01%)
Diabetes Mellitus	60/284 (21.13%)
Hypertension	137/284 (48.24%)
Anticholinergic Use	70/284 (24.65%)
Beta Agonist Use	94/284 (33.10%)

Bulkamid™ injection was associated with significant improvements in continence and lower urinary tract symptoms. Median daytime pad use decreased from 2.0 (IQR 0.0–3.0) pre‐procedure to 1.0 (IQR 0.0–1.5) post‐procedure (*p* < 0.001). There was a significant reduction in daytime pad usage 2 (IQR 0–3.0) to 1 (IQR 0–1.5) (median difference −1.5, *p* < 0.001). A post‑void residual (PVR) measurement was completed in 94% of patients pre‑procedure and 73% of patients post‑procedure (Table 2).

**Table 2:** Outcome measures.

Outcome measure	Pre‐Intervention (Median IQR)	Post‐Intervention (Median IQR)	Median difference (est.)	*p* a
Post Void Residual (mL)	5.0 (0.0–34.5)	0.0 (0.0–17.3)	−13.50	**< 0.001**
Number of Pads	2.0 (0.0–3.0)	1.0 (0.0–1.5)	−1.50	
UDI‐6 Q1: Frequent Urination	2.0 (1.0–3.0)	1.0 (0.0–1.0)	−1.00	
UDI‐6 Q2: Urgency Leakage	2.0 (1.00–3.0)	1.0 (0.0–2.0)	−1.50	
UDI‐6 Q3: Physical Activity	3.0 (3.0–3.0)	1.0 (0.0–2.0)	−2.00	
UDI‐6 Q4: Urine Leakage	3.00 (2.0–3.0)	1.0 (0.0–2.0)	−1.50	
UDI‐6 Q5: Emptying	1.00 (0.0–2.0)	0.0 (0.0–1.0)	−1.00	
UDI‐6 Q6: Pain Discomfort	1.00 (0.0–1.0)	0.0 (0.0–1.0)	−1.00	
Total UDI‐6	45.8 (33.3–58.3)	16.7 (8.3–32.3)	−22.93	

*Note:* Bold values indicated a *p*‐value that met the threshold of significance (< 0.05).

^a^ Wilcoxon signed‐rank test.

**Figure 1:**
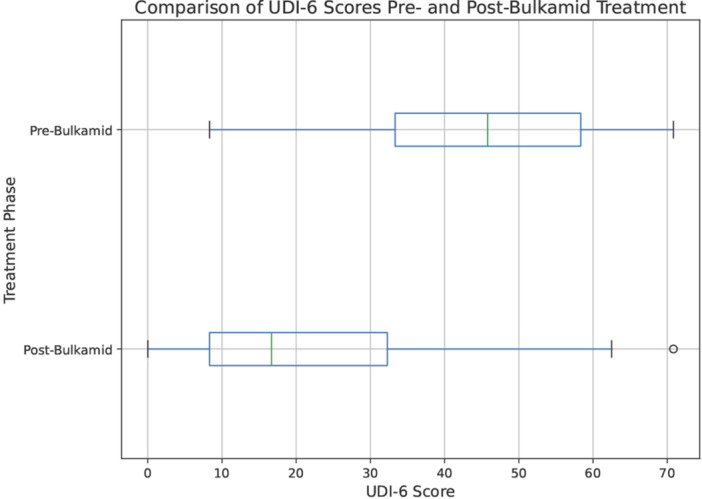
Comparison of UDI‐6 scores before and after Bulkamid treatment. Boxplots display the median, interquartile range (IQR), and range, with outliers shown as individual points.

The UDI‑6 completion rate was 31% pre‑procedure and 32% post‑procedure. UDI‐6 scores showed significant reductions across all measured domains. Total UDI‐6 scores decreased from a median of 45.8 (IQR 33.3–58.3) prior to treatment to 16.7 (IQR 8.3–32.3) post‐treatment (*p* < 0.001) (Figure 1) with a median time in‐between questionnaires of 5.85 months (IQR 3.29–13.81). (Table 2).

Forty‐seven patients (16.5%) required a second injection, at a median of 7.6 months (IQR 2.8–18.7) following the index procedure. Univariate analysis did not identify significant predictors of repeat injection, including age, BMI, SUI or MUI diagnosis, diabetes, hypertension, smoking status, or hysterectomy history.

Seventeen patients (6.0%) progressed to sling placement following Bulkamid™ injection with a median time to sling of 5.23 months (IQR 4.04–11.61). Younger age was significantly associated with sling progression (OR 0.95 per year, 95% CI 0.91–0.98, *p* = 0.007). The number of pads used during waking hours demonstrated a trend toward significance (*p* = 0.051). No other clinical or demographic predictors were identified (Table 3).

**Table 3:** Predictors of progression to sling^a^.

Characteristic	Odds ratio (OR)	95% Confidence interval	*p*
Age	0.95	0.91–0.98	**0.007**
AA Race	1.27	0.07–7.17	0.8
BMI	1.05	0.96–1.15	0.3
Smoking History			
Former	1.26	0.33–4.01	0.7
Current	2.35	0.12–14.7	0.4
Abdominal Leak Point Pressure	1.01	0.98–1.03	0.6
DO on UDS	1.22	0.06–10.1	0.9
Without Anesthesia	0.40	0.02–2.06	0.4
No. of pads prior to Bulkamid™	1.26	0.97–1.57	0.051
PVR prior to Bulkamid™	0.96	0.90–1.00	0.13

*Note:* Bold values indicated a *p*‐value that met the threshold of significance (< 0.05).

Abbreviations: AA, African American; DO, detrusor overactivity; PVR, post‐void residual; UDS, Urodynamic Studies.

^a^ Logistic Regression Model.

When comparing anesthesia type, patients without anesthesia had a lower post‐procedure UDI‐6 score for frequent urination (median 0.0 vs. 1.0, *p* = 0.010). No significant differences were observed between groups in score changes for frequent urination, urgency leakage, activity leakage, or other leakage (all *p* > 0.2).

Table 4 summarizes outcomes by anesthesia status. Decrease in pad use per day did not differ between groups (median 1.0 with anesthesia vs. 1.5 without anesthesia, *p* = 0.5). On UDI‐6 items, patients without anesthesia had lower post‐procedure UDI‐6 scores for frequent urination (median 0.0 vs. 1.0, *p* = 0.010), difficulty emptying (0.0 vs. 0.0, *p* = 0.021), and pain/discomfort (0.0 vs. 0.0, *p* = 0.014). No significant differences were observed for urgency leakage, activity leakage, or other leakage. Changes in individual UDI‐6 scores were not significantly different between groups (all *p* > 0.2). Median total post‐procedure UDI‐6 score was similar (16.7 without anesthesia vs 18.8 with anesthesia, *p* = 0.2), as was change in total UDI‐6 score (−25.0 vs. −20.8, *p* = 0.3). Median follow‐up time was longer in the no‐anesthesia group (13.4 vs. 6.2 months, *p* = 0.016).

**Table 4:** Comparison of outcomes for Bulkamid™ injection with versus without anesthesia.

Outcome measure	Anesthesia (*n* = 239)	No anesthesia (*n* = 45)	*p* a
Decrease in Pads per Day, Median (IQR)	1.00 (0.00–2.00)	1.50 (0.00–2.00)	0.5
Q1*: Frequent Urination (Post), Median (IQR)	1.00 (0.00–2.00)	0.00 (0.00–1.00)	**0.010**
Q1*: Change in Score, Median (IQR)	−1.00 (−2.00 to 0.00) (*n* = 62.00)	−2.00 (−2.00 to 0.00)	0.2
Q2*: Urgency Leakage (Post), Median (IQR)	1.00 (0.00–2.00)	1.00 (0.00–1.00)	0.4
Q2*: Change in Score, Median (IQR)	−1.00 (−1.00–0.00)	−1.00 (−2.00 to 1.00)	0.2
Q3*: Activity Leakage (Post), Median (IQR)	1.00 (0.00–2.00)	1.00 (1.00–1.00)	0.4
Q3*: Change in Score, Median (IQR)	−2.00 (−2.00 to −1.00)	−2.00 (−2.00 to −1.00)	0.9
Q4*: Other Leakage (Post), Median (IQR)	1.00 (0.00–2.00)	1.00 (1.00–2.00)	0.8
Q4*: Change in Score, Median (IQR)	−1.00 (−2.00 to −0.50)	−1.00 (−2.00 to 0.00)	> 0.9
Q5*: Difficulty Emptying (Post), Median (IQR)	0.00 (0.00–1.00)	0.00 (0.00–0.00)	**0.021**
Q5*: Change in Score, Median (IQR)	0.00 (−1.00–0.00)	0.00 (−1.00 to 0.00)	0.4
Q6*: Pain/Discomfort (Post), Median (IQR)	0.00 (0.00–1.00)	0.00 (0.00–0.00)	**0.014**
Q6*: Change in Score, Median (IQR)	0.00 (−1.00–0.00)	0.00 (−1.00 to 0.00)	0.6
Total UDI Score (Post), Median (IQR)	18.75 (8.33–37.50)	16.66 (10.42–20.83)	0.2
Total Change in UDI Score, Median (IQR)	−20.83 (−33.33 to −8.34)	−25.00 (−37.50 to −16.67)	0.3
Total Follow‐up Time (months), Median (IQR)	6.15 (1.05–15.85)	13.41 (1.15–25.87)	**0.016**
Months in‐between UDI Surveys (months)	7.07 (3.65–14.07)	2.76 (1.87–4.83)	**0.003**

*Note:* Bold values indicated a *p*‐value that met the threshold of significance (< 0.05).

^a^ Wilcoxon rank sum test.

* UDI‐6 question.

On multivariable logistic regression, undergoing Bulkamid™ injection without anesthesia was a significant predictor of progression to a second injection (OR 2.82, 95% CI 1.33–5.79, *p* = 0.005). Abdominal leak point pressure (OR 1.01, 95% CI 1.00–1.02, *p* = 0.088) and number of pads used prior to Bulkamid™ (OR 1.13, 95% CI 0.99–1.29, *p* = 0.057) demonstrated trends toward significance. Other factors, including age, race, BMI, smoking history, detrusor overactivity on urodynamics, and pre‐procedure post‐void residual, were not significantly associated with the need for a second injection (Table 5).

**Table 5:** Predictors of progression to 2nd injection^a^.

Characteristic	Odds ratio (OR)	95% Confidence interval	*p* a
Age	0.99	0.97–1.01	0.4
AA Race	0.63	0.10–2.34	0.5
BMI	0.99	0.93–1.04	0.7
Smoking History			
Former	1.38	0.67–2.75	0.4
Current	1.41	0.21–5.96	0.7
Abdominal Leak Point Pressure	1.01	1.00–1.02	0.088
DO on UDS	2.02	0.67–5.74	0.2
Without Anesthesia	2.82	1.33–5.79	**0.005**
No. of pads prior to Bulkamid™	1.13	0.99–1.29	0.057
PVR prior to Bulkamid™	0.99	0.98−1.00	0.15

*Note:* Bold values indicated a *p*‐value that met the threshold of significance (< 0.05).

Abbreviations: AA, African American; DO, Detrusor Overactivity; UDS, Urodynamic Studies; PVR, Post‐Void Residual.

^a^ Logistic Regression Model.

## 4 Discussion

In this single‐institution study of 284 women undergoing a Bulkamid™ injection performed by multiple surgeons, we observed significant improvements in both objective and patient‐reported measures of continence. Daytime pad use, and all UDI subdomains improved meaningfully pre vs post‐injection which are consistent with prior studies [3]. These findings reinforce the role of Bulkamid™ as a minimally invasive and effective treatment for SUI and MUI.

In our study, 16% of patients required a second injection at a median of 7.6 months, consistent with reported reinjection rates of 10%–30% in the literature. Most demographic and clinical factors—including age, BMI, comorbidities, and incontinence subtype—were not predictive of retreatment. However, anesthesia type was significant: patients treated without anesthesia had nearly threefold higher odds of requiring repeat injection (OR 2.82, 95% CI 1.33–5.79). This finding suggests that procedural setting and technique may influence durability, possibly due to differences in injection depth, visualization, or patient tolerance in the outpatient environment. Prior studies report satisfaction rates of 60%–85% at 12 months with reinjection rates of 20%–30%, though few specify anesthesia type [4, 8]. Smaller outpatient series have shown ~60%–70% cure or improvement at short‐term follow‐up with reinjection rates similar to mixed‐anesthesia cohorts, underscoring the need for more robust, stratified data [6, 7, 9].

Progression to sling in our cohort was uncommon, occurring in only 6% of patients. Younger age was a significant predictor, which may reflect greater functional demands and lower tolerance for persistent leakage among younger women [10]. While prior work by Faurie et al. identified pad use, but not age, as a short‐term predictor of Bulkamid™ success, our findings suggest that age may play a larger role in longer‐term durability [6]. In our cohort, daytime pad use showed a trend toward significance but did not reach statistical significance. Notably, although younger patients may prefer office‐based procedures, in our study age did not differ between anesthesia groups. The observed progression rate is relatively low and comparable to that seen after sling. In contrast, large database analyses demonstrate higher long‐term retreatment rates after bulking compared with sling (24.6% vs. 6.7% at 5 years), reflecting that bulking is often offered to patients for whom surgery is less suitable [11]. Importantly, Bulkamid™ provides the opportunity to individualize continence goals: for some patients, partial improvement is acceptable and avoids the risks of a sling, while others may ultimately require progression to surgery. Ultimately, treatment choice should be driven by shared decision‐making. Current data support sling surgery as the most effective option with the exception of older patients with anesthesia risk and younger women who wish to preserve fertility.

Strengths of this study include the inclusion of a large, contemporary cohort treated by multiple surgeons, which enhances the generalizability of our findings. We incorporated both objective clinical outcomes and validated patient‐reported outcome measures, allowing for a comprehensive assessment of treatment efficacy.

However, several limitations must be acknowledged. First, office‐based Bulkamid™ injections were performed earlier in our institutional experience, whereas subsequent procedures were performed in the operating room under anesthesia due to logistical and compensation changes. As a result, differences in outcomes may partly reflect temporal and learning curve effects rather than true differences attributable to the procedural setting. Additionally, the retrospective design of the study introduces a risk of selection bias and limits our ability to assess long‐term outcomes beyond the available follow‐up period. The variable survey completion rates also led to missing data in some subdomains of the UDI, which may impact the overall analysis. It is also important to interpret the reinjection timing in the context of our cohort's median follow‐up duration of 6.7 months. Many patients had not yet reached the window when repeat treatment is typically pursued. Because only a small proportion of patients ultimately underwent a second injection, the reported median reinjection time reflects only that small subgroup and does not represent the experience of the entire cohort. Additionally, many women choose not to pursue retreatment despite partial improvement, making reinjection rates difficult to interpret. Our study also does not include a control group comprised of patients who only underwent MUS, which prevents reported improvements from being contextualized in the broader landscape of SUI treatments. Finally, this study represents a single‐institution experience, and thus, our findings may not fully apply to all clinical settings or practices.

## 5 Conclusion

Bulkamid™ injection is a safe, effective, and minimally invasive treatment for stress and mixed urinary incontinence. It provides significant symptom improvement with low rates of repeat injection and sling conversion. Younger age predicted progression to sling; procedures performed without anesthesia predicted the likelihood of repeat injection. Bulkamid™ remains a valuable option for women seeking alternatives to sling surgery.

## Funding

The authors have nothing to report.

## Ethics Statement

This study was approved by the Institutional Review Board at the University of Texas Southwestern Medical Center (IRB# STU‐2024‐1125).

## Consent

Informed consent was waived due to the retrospective nature of the study, as approved by the IRB.

## Conflicts of Interest

The authors declare no conflicts of interest.

## Data Availability

The datasets generated and analyzed during the current study are available from the corresponding author on reasonable request.
